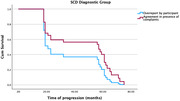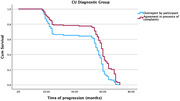# Agreement between self‐ and informant‐reports of cognitive complaints in predicting time to an objective cognitive worsening in Cognitively Unimpaired (CU) and with Subjective Cognitive Decline (SCD) participants: Results from the CompAS longitudinal study

**DOI:** 10.1002/alz.087070

**Published:** 2025-01-03

**Authors:** Lucía Pérez‐Blanco, Alba Felpete, Ana Nieto‐Vieites, Cristina Lojo‐Seoane, Carlos Spuch, Jose M. Aldrey, Onésimo Juncos‐Rabadán, Arturo X. Pereiro Rozas

**Affiliations:** ^1^ Department of Developmental and Educational Psychology, University of Santiago de Compostela, Santiago de Compostela Spain; ^2^ Applied Cognitive Neuroscience and Psychogerontology group, Health Research Institute of Santiago de Compostela (IDIS), Santiago de Compostela Spain; ^3^ Galicia Sur Health Research Institute (IISGS), Vigo Spain; ^4^ Health Research Institute of Santiago (IDIS), Santiago de Compostela Spain

## Abstract

**Background:**

The importance of coincidence of cognitive complaints between participants without objective impairment and their informants in predicting progression remains unclear (Nosheny et al, 2022). Our objective was to determine whether agreement in dyadic reporting at baseline can predict survival time to progression to MCI or dementia.

**Method:**

A sample of 145 participants from the CompAS Study was included in a survival analysis. They were identified at baseline as CU (N = 115) and SCD (N = 30) and re‐diagnosed as CU, SCD, MCI, or dementia from 17 to 76 months of follow‐up. ‘Agreement’ in the presence of complaints was computed depending on the informant and participant QAM scores (range: 7‐35) at baseline were above or below a cut‐off point (i.e., 10‐11): 1) Overreport by participant (i.e., QAM scores above the established cut‐off for participant and below for informant); 2) Agreement in presence of complaints (i.e., QAM total scores of both coincide in being above the established cut‐off point). The other two categories (i.e., Overreport by informant; Agreement in absence of complaints) were discarded because there were almost no cases. We performed a Cox proportional hazards regression model (no cases censored). The event was defined as stability when the baseline diagnosis remained stable over the follow‐ups and worsening when a change to MCI or dementia. ‘Agreement’ was introduced as a covariate. Two groups (CU; SCD) identified at baseline entered the model as strata.

**Result:**

The model was statistically significant [model fit: *‐2 Log Likelihood* = 1029.810, *χ^2^* = 9.721, *df* = 1, *p*<.002]. ‘Agreement’ significantly predicts time survival [*β* = ‐.471, *SE* = .161, *Wald* = 8.569, *p* < .003] and *HR* = .575 (95% CI = .404‐.817) indicated that the risk of worsening per month was 42.5% (1‐.575 = .425) smaller for the ‘Agreement in presence of complaints’ than for the ‘Overreport by participant’ in both CU and SCD groups (Figures 1 and 2).

**Conclusion:**

Our results suggest that compared to the ‘Overreport by participant’, the dyadic agreement in the presence of complaints decreases the risk of worsening over time. These results could have to do either with a greater awareness of one’s own difficulties or with the beneficial effect of agreeing on its detection with informants in pre‐symptomatic stages.